# Double-Strip Array-Based Metasurfaces with BICs for Terahertz Thin Membrane Detection

**DOI:** 10.3390/mi15010043

**Published:** 2023-12-25

**Authors:** Yanchun Shen, Jinlan Wang, Hongyu Sheng, Xiaoming Li, Jing Yang, Hongmei Liu, Dejun Liu

**Affiliations:** 1 College of Information Engineering, Guangzhou Railway Polytechnic, Guangzhou 511300, China; wangjinlan@gtxy.edu.cn (J.W.); ming00ming@126.com (X.L.); yangjing@gtxy.edu.cn (J.Y.); liuhongmei@gtxy.edu.cn (H.L.); 2 College of Robotics, Beijing Union University, Beijing 100101, China; jzw@buu.edu.cn; 3 Department of Physics, Shanghai Normal University, Shanghai 200234, China

**Keywords:** terahertz detection, metasurface, bound states in the continuum, high quality

## Abstract

A double-strip array-based metasurface that supports the sharp quasi-bound states in the continuum (quasi-BICs) is demonstrated in terahertz regions. By tuning the structural parameters of metal strips, the conversion of BICs and quasi-BICs is controllable. The simulated results exhibit an achieved maximum Q-factor for quasi-BICs that exceeds 500, corresponding to a bandwidth that is less than 1 GHz. The optical response of quasi-BICs is mainly affected by the properties of substrates. Resonant frequencies decrease linearly with increasing refractive index. The bandwidth of quasi-BICs decreases to 0.9 GHz when *n* is 2.2. The sharp quasi-BICs are also sensitive to changes in material absorption. Low-loss materials show higher Q-factors. Thus, the selection of a suitable substrate material will be beneficial in achieving resonance with a high Q value. The sensitivity of DSAs for molecules is assessed using a thin membrane layer. The DSAs show high sensitivity, which achieves a frequency shift of 70 GHz when the thickness of the membrane is 10 μm, corresponding to a sensitivity of 87.5 GHz/RIU. This metasurface with sharp quasi-BICs is expected to perform well in THz sensing.

## 1. Introduction

Bound states in the continuum (BICs) describe exotic localized eigenstates embedded in the continuous spectrum, which is initially proposed by von Neumann and Wigner in 1929 [1]. BICs eliminate radiation loss and thus allow for resonances with an infinite quality factor (Q-factor), showing high potential in the field of lasing [2], biosensing [3], imaging [4], and nonlinear harmonic generation [5]. In terahertz (THz) regions, BICs are widely applied in sensing applications. By changing the incident angle or structural parameters, BICs can be converted to quasi-BICs with high Q-factors. Resonances with high Q-factor show an enhanced field, benefitting light-matter interactions. Metasurfaces with periodic meta-atoms are perfect platforms to manipulate BICs [6,7,8,9,10]. Recently, different types of metasurfaces that consist of various materials have been proposed for BICs manipulations [11,12,13,14,15,16]. Dielectric metasurfaces are excellent candidates for high Q-factor BICs due to their low material losses [17,18,19]. The Q-factor of dielectric metasurfaces can be increased by up to 10^4^ by breaking the symmetries of metasurfaces [20]. The Q-factor measured by THz-TDS is lower than 100. Furthermore, dielectric metasurfaces confine the enhanced field inside the structure, exhibiting weak interactions between the field and analyte layers. Metal hole array (MHA)-based metasurfaces have been reported for the detection of protein molecules because of the induced surface plasmon polaritons (SPPs) with the surface-enhanced field [21,22]. For the printed horseradish peroxidase with 500 pg/mm^2^, due to the change in refractive index, the transmission dip is red-shifted. MHAs need oblique incident waves to excite sharp resonance, which is complicated and limited in THz sensing [21]. In order to address this problem, Liu, D. et al. have introduced quasi-BICs for full metal structures [14,23]. By tuning the structural parameter, a maximum Q-factor of 102 of quasi-BICs can be measured. Subsequently, Wang et al. used such kinds of BICs metasurfaces for polymer membrane sensing [24]. A sensitivity of 151 GHz/RIU for 50-μm polymer was obtained, higher than bulky dielectric substrate metamaterials. To further improve the sensitivity of quasi-BICs sensors, a metasurface based on four-hole arrays was proposed, which realizes a polarization-independent quasi-BIC and a high Q-factor exceeding 2000 after optimization [25]. For a 25 µm-thick polyimide membrane, the measured results exhibit that the frequency shift is 97.5 GHz. The full metallic structure shows high Ohmic loss, hindering the generation of high Q-factor quasi-BICs. Thus, metal-dielectric-based metasurfaces with about 200 nm thick metal layers have been demonstrated. Such metasurfaces reduce the Ohmic loss from thick metal. Y. K. Srivastava et al. reported an asymmetric split ring on a low refractive index substrate with quasi-BICs, which allows the detection of an analyte with a thickness of 7 nm at terahertz frequencies [26]. The analyte layer is coated on the metasurface, which is difficult to clean after sensed. Thus, reusable sensors based on BICs metasurfaces are in urgent need. In addition, the effect of material properties of substrates such as thickness and absorption on the Q-factor of resonances is not clear, which warrants further analysis.

Here, the quasi-BICs that are supported by the metasurface consisting of double-strip arrays are studied at THz frequencies. The transformation between BICs and quasi-BICs can be controlled by changes in the length of one metal strip. The achieved maximum Q-factor exceeds 500, corresponding to a bandwidth that is less than 1 GHz. We thoroughly analyzed the induced quasi-BICs by using the Fano fitting and electric field vectors; results show that the quasi-BICs manifests itself as a type of Fano resonance when structural symmetry breaks down. Differing from traditional resonances of WA with the same profile, the field vector direction of quasi-BICs on the strip surface is opposite, showing a phase difference of 180°. The role of substrate materials on resonant responses of quasi-BICs is thoroughly discussed. The bandwidth of quasi-BICs is influenced by the substrate thickness. The narrowest bandwidth (1.8 GHz) can be realized when the substrate thickness is 50 μm and 60 μm. Refractive indices of the substrate also dramatically change the optical response of the metasurfaces. Resonant frequencies decrease linearly with increasing refractive index. The bandwidth of quasi-BICs decreases to 0.9 GHz when *n* is 2.2. The sharp quasi-BICs are sensitive to the changes in materials absorption. Low-loss materials show higher Q-factors. Thus, suitable substrate material selection will benefit the achieving high Q-factor resonance. Finally, the sensitivity of proposed DSAs for molecules is assessed using the BSA layer. The DSAs show high sensitivity, achieving a frequency shift of 70 GHz when the thickness of BSA is 10 μm, corresponding to a sensitivity of 87.5 GHz/RIU. Thus, this metasurface with sharp quasi-BICs is expected to perform well in THz sensing.

## 2. The Design of Double-Strip Arrays

**Figure 1:**
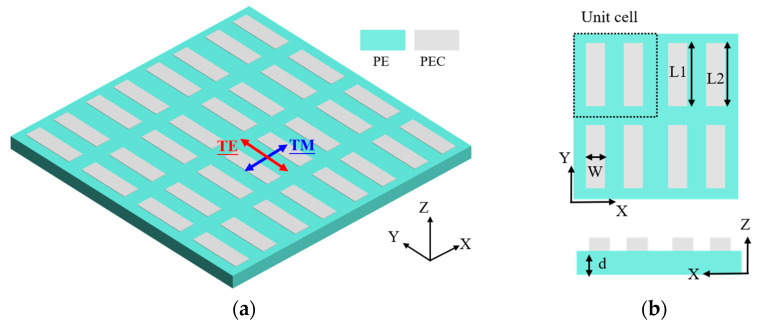
(**a**) Configuration of double-wire arrays; (**b**) the unit cell of double-strip arrays.

The 3D configuration of proposed metasurfaces based on double-strip arrays (DSAs) can be seen in Figure 1. Differently from the previous reference with thick quartz substrate [27], the proposed DSAs consists of two metal strips that cover a flexible ultrathin substrate. The thin substrate can reduce material absorption and improve Q-factors [28]. This flexible substrate is polyethylene (PE) (*n* = 1.52 at 1 THz) [29]. The thickness of the substrate is *d* with a value of 50 μm. Here, numerical results are carried out through CST Studio Suite. The metal conductivity strongly affects the profile of resonances when the metasurface has extremely low structural asymmetry [30]. In the simulation, the metal is set as a perfect electron conductor (PEC) with a thickness of 200 nm, and the material absorption of PE is not considered. As shown in Figure 1b, each double-strip array is considered as a unit and the period of the array is set as Λ. The metal strip has a width of w. The two strips have a respective length of L1 and L2. What follows are the detailed parameters: the period is Λ = 260 μm; the width of strips is w = 60 μm; the length of strips is L1 = 200 μm. L2 is selected as a variable parameter, which determines the structural states (symmetry or asymmetry). Such states provide a route for the conversion of perfect BICs and quasi-BICs [31]. For transverse electric (TE) modes, the electric field is transverse to the direction of propagation while the magnetic field is normal to the direction of propagation. For transverse magnetic (TM) modes, the magnetic field is transverse to the direction of propagation while the electric field is normal to the direction of propagation. In the CST simulation, the electric field of the TE and TM modes is, respectively, perpendicular to the X- and Y-axes, as shown in Figure 1a.

## 3. Simulation Results and Analysis

**Figure 2:**
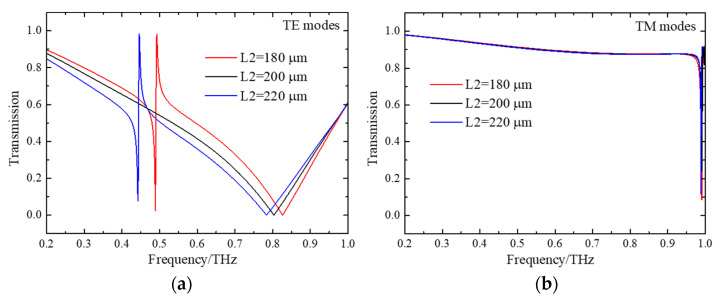
(**a**) For TE waves, the transmission spectra of DSAs with different L2. (**b**) For TM waves, the transmission spectra of DSAs with different L2.

Figure 2 shows the transmission spectra at normal incidence of TE and TM modes for DSAs with various structural parameters. The DSAs are symmetric in the X- and Y-directions when L1 is equal to L2. For a periodic structure, the free-space frequency *f_WA_* satisfies the WA condition. Thus, it shows a transmission minimum. The formulation of WA is expressed as *f_WA_* = C/(Ʌ*n_eff_), in which *n_eff_* is the effective refractive index. In Figure 2a, a transmission dip is found in the spectrum for TE modes, which can be termed a Wood anomaly (WA) [32,33]. Other resonance is not induced because the symmetry state of DSAs is maintained. The corresponding spectra are different from the complementary structure of double-slit arrays in the previous reference [14]. As L2 increases or decreases, a sharp resonant dip appears because the structural symmetry is broken. When L2 = 180 μm, a resonance with a sharp profile is induced at 0.49 THz (red line). As L2 increases to 220 μm, the sharp resonance shifts to 0.44 THz (blue line). Such resonance performs as a typical feature of Fano resonance with an asymmetric line shape [21,34]. But for TM modes, the structural symmetry in the X-direction is sustained, and, thus, sharp resonance is not induced. As proved in previous references [35,36,37], perfect BICs are a dark mode that happen only at L1 = L2, which convert to the bright mode of quasi-BICs with a high Q value when the L2 is different from L1 due to the broken structural symmetry. In other words, the changes in structural symmetry cause the emergence of quasi-BICs.

**Figure 3:**
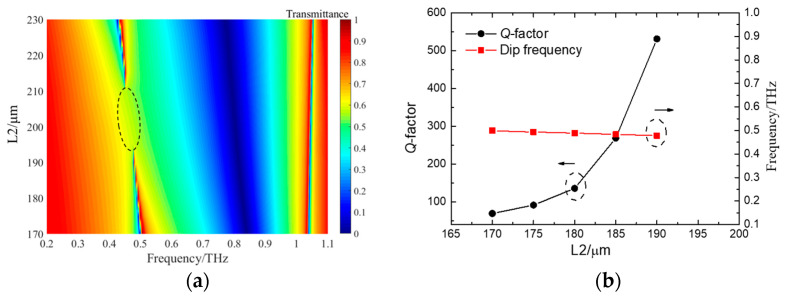

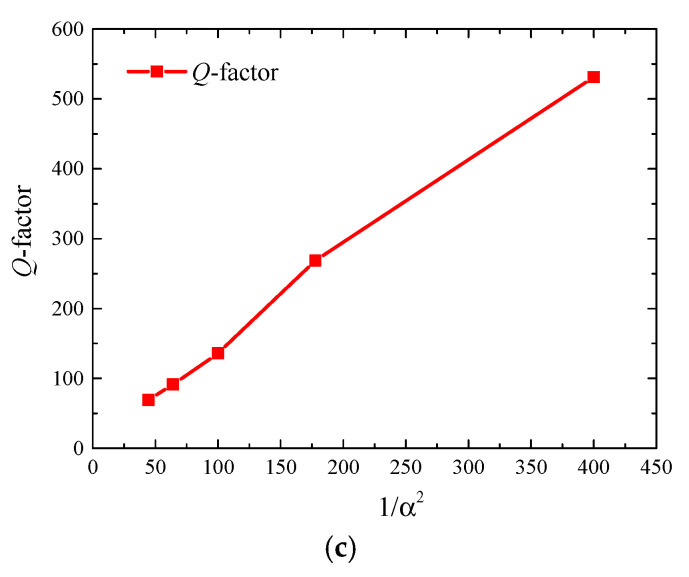
(**a**) The transmission spectra of double-strip arrays with different L2s. (**b**) The corresponding Q-factor and resonant frequency with L2 changes. (**c**) The relation between Q-factor and asymmetric parameter α.

To further confirm the existence of such BICs under TE wave incidences, the map of transmission spectra of the DSAs versus different L2s is calculated and shown in Figure 3a. Clearly, the sharp resonant profile vanishes when L2 is close to the value of 200 μm, which means that the BICs are almost decoupled to the incidence waves [37]. The result shows that the ideal BIC appears at 0.485 THz, which can be adjusted by changing the material properties of substrates [31]. With L2 increases, the quasi-BICs appear and redshift. Quasi-BICs bandwidths widen, corresponding to the decreases of Q-factors. Similarly, when L2 decreases, the induced quasi-BICs move to higher frequencies. These results describe the characteristics of BICs. Such BICs are also called symmetry-protected BICs [38]. Once the structural symmetry is broken, bound states of quasi-BICs change to radiation modes [35]. Figure 3b reveals the alteration of L2 results in the changes of quasi-BICs. One can see that the resonant profiles can be modified by varying the structural parameters of L2. As L2 increases from 170 μm to 190 μm, the corresponding Q-factor shows an exponential growth. It is clear in Figure 3b that the simulated maximum Q-factor exceeds 500, corresponding to a bandwidth less than 1 GHz. However, it is difficult to realize ultrahigh Q-factors in experiments because of the limitation of fabrication conditions for small asymmetric parameters. In addition, the low resolution of THz-TDS restricts the observation of high Q-factors. The broken symmetry also changes the volume of metasurfaces, resulting in slight changes of resonant frequencies, as shown in Figure 3b. The changes in L2 refer to a changed asymmetric factor *a =* (|(L2 − L1)/L1|). The corresponding Q-factor satisfies the formula Q∝1/*a*^2^ [14,21,39]. Therefore, the smaller the asymmetric factor, the larger the Q-factor [Figure 3c].

**Figure 4:**
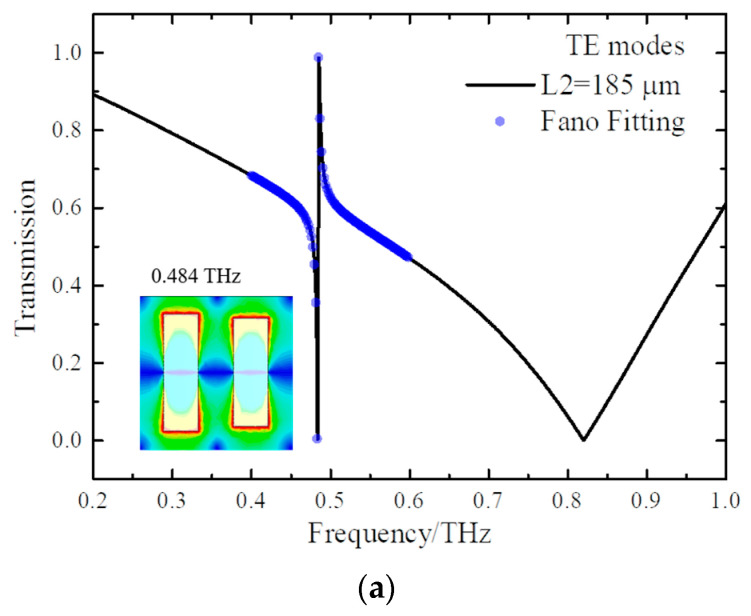

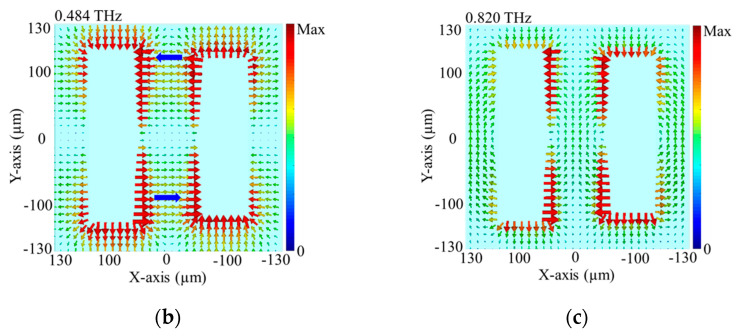
(**a**) The transmission spectrum of DSAs when L2 is 185 μm, where the inset figure is the field distribution of 0.484 THz; (**b**) field vectors of 0.484 THz for quasi-BICs; (**c**) field vectors of 0.820 THz for WA.

In Figure 4a, a sharp quasi-BICs is depicted in the transmission spectrum, where L2 is 185 μm. Here, the structural state of DSAs is broken. Thus, the quasi-BICs with a Q-factor of 286.6 can be observed at 0.484 THz. This resonance originating from the symmetry breaking would be fit by the Fano formula [23],

(1) $$T={\left|a_{1}+ja_{2}+\frac{b}{\omega -\omega_{0}+j\gamma }\right|}^{2}$$

This means that the quasi-BICs manifest themselves as a type of Fano resonance as the structural symmetry is broken [40,41]. It can be seen in Figure 4a, for a field distribution at 0.484 THz, that the strong field is located at the edge of metal strips, which is sensitive to material changes. To better understand the physical mechanism of quasi-BICs, the electric field vector distribution is simulated. The resonance of WA at 0.820 THz is selected as an example for comparison. At 0.484 THz, the surface field is excited and concentrated at the upper and lower boundaries of the metal strip and the edge of the gap between the two strips. The vector directions on the surface of the two strips are opposite, showing the phase difference of 180°, which indicates the quasi-BICs are formed. But at 0.820 THz of WA, the surface vector directions are the same. Thus, the profile of quasi-BICs differs from the traditional resonances of WA.

**Figure 5:**
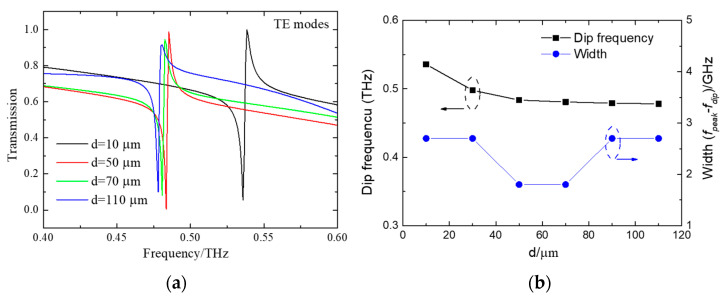
(**a**) The calculated transmission spectra for different PE thicknesses; (**b**) the changing trend of resonant frequency and width with the alters of PE thickness *d*.

The properties of substrate materials have a giant effect on the performance of quasi-BICs [28,42,43,44,45]. Correctly selecting the substrate with suitable parameters is advantageous for obtaining high Q-factor modes. The substrate effect appears due to the finite dielectric contrast between the superstrate and substrate claddings [42]. Here, the relationship between PE thickness and the profile of quasi-BICs is analyzed. The calculated transmission spectra with various substrate thicknesses under TE mode incidence is depicted in Figure 5a. It is clearly seen that the resonance shifts as the PE thickness, *d*, changes. In addition to the spectral shift induced by the PE, a dramatic change in resonant width is found. Figure 5b summarizes the resonant frequency and width as a function of PE thickness *d*. With the increasing PE thickness, the resonance moves toward low frequencies. For example, *d* = 10 μm corresponds to a resonance at 0.540 THz, shifting to 0.455 THz once *d* increases to 110 μm. The bandwidth of quasi-BICs is also affected by the substrate, which decreases first and then increases as the PE thickness increases. The narrowest bandwidth of 1.8 GHz can be realized when the thickness is 50 μm and 60 μm. The bandwidth increases to 2.7 GHz when the PE thickness increases to 100 μm. Thus, selecting a suitable substrate thickness benefits achieving a high Q-factor resonance.

**Figure 6:**
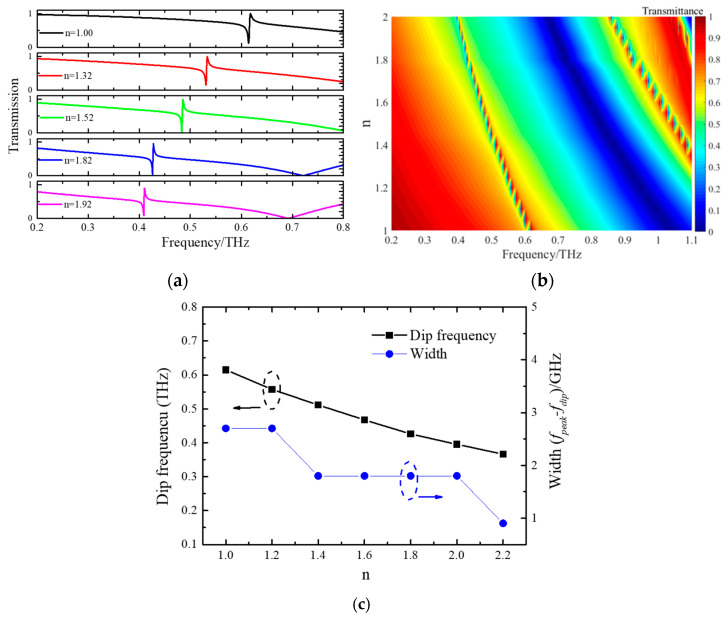
(**a**) The calculated spectra of DSAs for different *n*; (**b**) the spectral map for different *n*; (**c**) the relationship between *n* and the resonant frequency and bandwidth.

The above results proved that this metasurface can achieve high Q-factor quasi-BICs when the structural symmetry is broken and thus its potential to be used as a biosensor [24,25,28,46]. To verify the functionality of DSA-based sensors, we have simulated the transmission spectra of DSAs with different substrate refractive indices (*n*). The thickness of the substrate is fixed as 50 μm and L2 is set as 185 μm. As shown in Figure 6a, as *n* increases, resonant peaks in the transmission spectrum gradually shift towards low frequencies. When *n* equals 1.92, the resonant dip changes to 0.415 THz. This suggests that the transmission dip position is significantly influenced by changes in the substrate’s refractive index. It also means that the resonance peaks will be sensitive to biological samples with different refractive indices [11,47,48]. For a clearer visual representation of how the refractive index shifts the resonant dip, a spectral map of the refractive index and resonance frequency is present in Figure 6b. The figure clearly illustrates the resonance peak shift. Meanwhile, the resonant bandwidth is also reduced as the refractive index of substrates increases. To learn more about how the substrate refractive index affects resonance characteristics, we have plotted the curve of resonant frequency and bandwidth with different *n*. In Figure 6c, with *n* increasing, the resonant frequency shows a linear decline due to its changed dispersion. The frequency of the resonant dip appears at 0.366 THz while *n* equals 2.2. However, the quasi-BICs bandwidths remain unchanged with a value of 1.8 GHz when *n* is altered from 1.4 to 2.0. It decreases to 0.9 GHz when *n* changes to 2.2. Thus, the optical response of the metasurfaces is drastically altered by the substrate’s refractive index.

**Figure 7:**
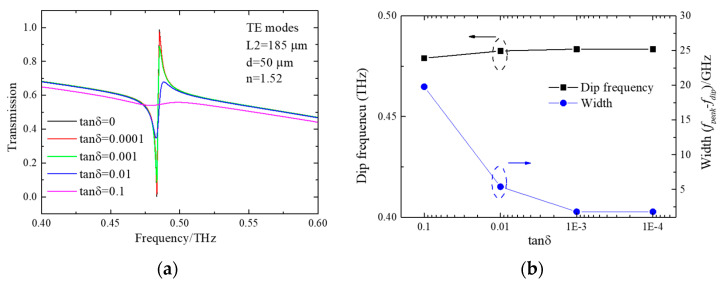
(**a**) The calculated spectra with different loss tangent; (**b**) resonant frequency and bandwidth of quasi-BICs as a function of tanδ.

Sharp quasi-BICs are sensitive to changes in materials such as absorption and refractive index [14,23,25,35]. In full metal-based metasurfaces, high conductivities show higher Q-factors [14,23,25]. To further reveal the effect of the material loss of the substrate on quasi-BICs, transmission spectra with different loss tangents have been simulated and performed. Parameters for structures are chosen to support sharp quasi-BICs. Here, L2 is selected as 180 μm and *d* is set as 50 μm. As seen in Figure 7a, the resonance becomes broad as the tanδ increases. It means that the Q-factor is reduced. Resonant frequency and bandwidth of quasi-BICs are summarized and shown in Figure 7b. Transmission spectra results show that material absorption does not significantly alter the locations of resonances [49,50]. But resonant bandwidth nonlinear decreases as the loss tangent increases. This confirms that the quasi-BICs are indeed sensitive when the loss of substrate is changed.

**Figure 8:**
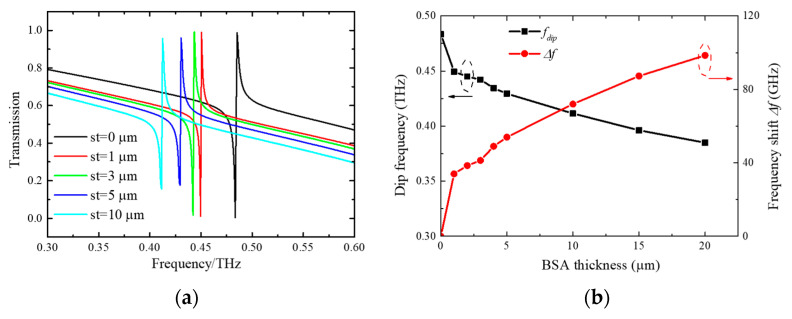
(**a**) The calculated spectra with different BSA thicknesses (*st*); (**b**) the summary of resonant frequency and frequency shift for different thicknesses (*st*).

THz sensing technology is beneficial for thin-film detection with a small volume of samples. A serum albumin protein of bovine serum albumin (BSA) could prevent nonspecific binding sites from being used during protein–protein interactions [51]. Thus, BSA is frequently utilized as a THz biomarker for biological detection. Here, the BSA membrane is selected to assess the performance and potential of the DSAs-based sensors [52,53]. BSA has a refractive index of 1.8 in 0.2–1.2 THz [51]. Our proposed structure is proven to be sensitive to detect the molecular layer with various thicknesses. Putting the analyte layer on the metasurface without processing, there is an air gap between the analyte layer and the metasurface [24]. In reference [26], the detection material is put on the metasurface-based sensors. Thus, such metasurfaces cannot be reused. In reference [25], alcohol is used to eliminate the air gap between the metasurface and the sensed membrane. After the alcohol evaporates, the membrane sticks tightly to the metasurface. Here, in the simulation, we do not consider that air gap. As seen in Figure 8a, the calculated spectra of DSAs with various thicknesses (*st*) of the BSA layer are presented. Numerical results reveal that small analyte thickness changes can result in a clear spectral shift. A 1 μm-thick BSA layer results in a frequency shift of 34 GHz. As the BSA thickness increases, the quasi-BICs further move to lower frequencies. In Figure 8b, we have summarized the resonant frequency and frequency shift with different BSA thicknesses. The proposed metasurface is sensitive to the attached molecular layers due to the enhanced electromagnetic field on the metal surfaces [54,55]. The strong field–analyte interaction results in large resonant frequency shifts. As the BSA layer thickness increases, the frequency shift Δ*f* shows a nonlinear trend. The frequency shift Δ*f* is 70 GHz when the thickness of the BSA is 10 μm, which corresponds to a sensitivity (S = Δ*f*/Δ*n*) of 87.5 GHz/RIU. The realization sensitivity is higher than that of full metal structures (60.6 GHz/RIU) [25]. Such metasurfaces can be used for microfluidic sensing because of their high sensitivity [56].

## 4. Conclusions

We have investigated the optical response of quasi-BICs in a metasurface consisting of double-strip arrays. The transformation between BICs and quasi-BICs can be controlled by changes in the length of one metal strip. The achieved maximum Q-factor exceeds 500, corresponding to a bandwidth that is less than 1 GHz. We have thoroughly analyzed the induced quasi-BICs by using the Fano fitting and electric field vectors. Results show that the quasi-BICs manifest themselves as a type of Fano resonance once the symmetry is broken. Differing from traditional resonances of WA with the same profile, the field vector direction of quasi-BICs on the strip surface is opposite, showing a phase difference of 180°. More discussion is given to the impact of substrate on the performance of quasi-BICs. The thickness of the substrate has a great impact on the bandwidth of quasi-BICs. The narrowest bandwidth of 1.8 GHz can be realized when the substrate thickness is 50 μm and 60 μm. The optical response of the metasurfaces is also significantly altered by the substrate’s refractive index. The resonant frequency shows a linear decline when *n* increases. The bandwidth of quasi-BICs decreases to 0.9 GHz when n is 2.2. The sharp quasi-BICs are sensitive to the changes in absorption of materials. Low-loss materials show higher Q-factors. Thus, suitable substrate material selection will benefit the achieving high Q-factor resonance. Finally, the BSA layer is used to assess DSA sensitivity. The DSAs show high sensitivity, achieving a frequency shift of 70 GHz when the thickness of BSA is 10 μm, corresponding to a sensitivity of 87.5 GHz/RIU. Thus, this metasurface with sharp quasi-BICs is expected to perform well in THz sensing.

## Data Availability

Upon reasonable request, Yanchun Shen, the corresponding author, will provide the data that support the study’s conclusions.
